# Modulation of Neurturin Expression by Lumbosacral Spinal Stenosis, Lifestyle Factors, and Glycemic Dysregulation

**DOI:** 10.3390/biomedicines13051102

**Published:** 2025-05-01

**Authors:** Małgorzata Sobańska, Dawid Sobański, Rafał Staszkiewicz, Paweł Gogol, Damian Strojny, Tomasz Pawłaszek, Werner Dammerman, Beniamin Oskar Grabarek

**Affiliations:** 1 Department of Neurosurgery, Szpital sw. Rafala in Cracow, 30-693 Cracow, Poland; drdsobanski@gmail.com; 2 Collegium Medicum, WSB University, 41-300 Dabrowa Gornicza, Poland; rafalstaszkiewicz830@gmail.com (R.S.); drpawelgogol@gmail.com (P.G.); drstrojnyds@gmail.com (D.S.); tomaszpawlaszek84@gmail.com (T.P.); bgrabarek7@gmail.com (B.O.G.); 3 Department of Neurosurgery, 5th Military Clinical Hospital with the SP ZOZ Polyclinic in Krakow, 30-901 Krakow, Poland; 4 Department of Neurosurgery, Faculty of Medicine in Zabrze, Academy of Silesia, 40-555 Katowice, Poland; 5 Department of Anesthesiology and Intensive Care, Our Lady of Perpetual Help Hospital in Wołomin, 05-200 Wołomin, Poland; 6 Department of Trauma and Orthopedic Surgery, Our Lady of Perpetual Help Hospital in Wołomin, 05-200 Wołomin, Poland; 7 Pain Treatment Clinic, Our Lady of Perpetual Help Hospital in Wołomin, 05-200 Wołomin, Poland; 8 Department of Neurology, New Medical Techniques Specjalist Hospital of St. Family in Rudna Mała, 36-060 Rzeszow, Poland; 9 Institute of Health Care, National Academy of Applied Sciences in Przemyśl, 37-700 Przemysl, Poland; 10 Center of Internal Medicine II, University Hospital Brandenburg, 03048 Brandenburg, Germany; werner.dammermann@mhb-fontane.de; 11 Faculty of Health Sciences Brandenburg, Brandenburg Medical School Theodor Fontane, 16816 Neuruppin, Germany

**Keywords:** lumbosacral spinal stenosis, neurturin, lifestyle, diabetes

## Abstract

**Background/Objectives**: Lumbosacral spinal stenosis (LSS) is a degenerative condition characterized by narrowing of the spinal canal and associated neuropathic pain. While mechanical compression is well-characterized, the molecular mechanisms contributing to symptom severity remain poorly understood. Neurturin (NRTN), a member of the glial cell line-derived neurotrophic factor family, has emerged as a potential mediator of neural plasticity and nociception, but its role in spinal stenosis is largely unexplored. **Methods**: We analyzed *NRTN* mRNA and protein expression in ligamentum flavum samples from 96 patients undergoing surgery for LSS and 85 non-degenerative postmortem controls. Quantification was performed using real-time quantitative polymerase chain reaction (RT-qPCR), enzyme-linked immunosorbent assay (ELISA), Western blotting, and immunohistochemistry. Pain severity Visual Analog Scale (VAS), body mass index (BMI), diabetes, smoking, and alcohol use were assessed as modulators of NRTN expression. **Results**: NRTN expression was significantly elevated in LSS patients versus controls at both transcript and protein levels (*p* < 0.05). NRTN levels positively correlated with pain intensity (VAS; ANOVA *p* = 0.032 for mRNA, *p* = 0.041 for protein). Multivariate regression identified BMI (β = 0.50, *p* = 0.015) and diabetes (β = 0.39, *p* = 0.017) as independent predictors of increased NRTN expression. Alcohol use also showed a positive association (*p* = 0.046), while smoking showed no significant independent effect. **Conclusions**: Neurturin is upregulated in ligamentum flavum tissue from LSS patients and correlates with pain severity and metabolic risk factors. These findings suggest NRTN as a potential biomarker and therapeutic target in degenerative spine disease. Further longitudinal and mechanistic studies are warranted to elucidate its role in chronic pain and neuroinflammation.

## 1. Introduction

Lumbosacral (L/S) spinal stenosis is a common degenerative condition characterized by narrowing of the spinal canal in the lumbar region, resulting in mechanical compression of neural and vascular structures. Clinically, it presents with lower back pain radiating to the buttocks and lower limbs, often accompanied by neurogenic claudication, which presents as discomfort during standing or walking, relieved by sitting or spinal flexion [1,2,3].

The L4/L5 level is most frequently affected, followed by L3/L4 and L2/L3, with occasional involvement of L5/S1 and L1/L2 [4].

While conservative therapies such as physiotherapy, pharmacologic management, and lifestyle modification are often effective in early stages, surgical decompression becomes necessary when neurological deficits progress or pain becomes refractory [5]. Standard surgical management includes laminectomy with resection of hypertrophied ligamentum flavum—a structure increasingly recognized not only as a passive mechanical contributor but also as an active participant in the disease process. Recent studies have highlighted that beyond structural impingement, lumbar spinal stenosis involves molecular dysregulation, including inflammatory and neurotrophic signaling within the ligamentum flavum [6,7]. Neurotrophic factors, long known for their role in neuronal survival and regeneration, have emerged as modulators of pain, inflammation, and tissue remodeling [8]. These factors are classified into three major families: classical neurotrophins, neuropoietic cytokines, and glial cell line-derived neurotrophic factor ligands (GFLs). Among GFLs, neurturin (NRTN) has drawn attention due to its roles in supporting peripheral neuron survival, modulating sensory function, and facilitating tissue adaptation following injury [9,10,11]. NRTN is a glycosylated protein structurally related to GDNF, produced as an inactive precursor that undergoes proteolytic processing to generate a mature, biologically active form [12]. This active form binds to GFRα2, initiating a signaling cascade via the RET tyrosine kinase receptor that activates ERK/MAPK, PI3K/Akt, and other survival and growth pathways [13,14,15,16,17]. While NRTN’s role in neural maintenance is well established, its potential involvement in degenerative spinal disorders, particularly in the context of ligamentum flavum hypertrophy, remains unexplored. To date, no comprehensive studies have examined how demographic variables (e.g., sex), metabolic status (e.g., BMI and diabetes), and lifestyle factors (e.g., smoking and alcohol use) influence NRTN expression in patients with lumbosacral spinal stenosis. Addressing this gap, the present study investigates the expression of NRTN at the mRNA and protein levels in ligamentum flavum tissue and evaluates its association with clinical pain severity and selected systemic risk factors. This approach aims to clarify whether NRTN could serve as a biomarker or therapeutic target in degenerative spine pathology.

Addressing this knowledge gap, the present study aimed to quantify NRTN expression at the mRNA and protein levels in ligamentum flavum tissues from patients with lumbosacral spinal stenosis and to evaluate its associations with pain severity and systemic risk factors, including demographic, metabolic, and lifestyle variables. By doing so, this study sought to determine whether NRTN could serve as a potential biomarker or therapeutic target in the context of degenerative spinal pathology.

## 2. Materials and Methods

This study expands on findings from previous research conducted by our team [18,19].

### 2.1. Characteristics of Study Group

The clinical cohort comprised 96 individuals diagnosed with lumbosacral (L/S) spinal stenosis who qualified for extended fenestration and foraminotomy surgery. The group included 46 women (48%) and 50 men (52%), with a mean age of 68.3 ± 2.4 years. Diagnosis was established through comprehensive clinical assessment and MRI imaging using 3 mm and 4 mm slice protocols in multiple planes. Lifestyle information, such as tobacco use and alcohol consumption, was self-reported; however, no quantification of frequency or volume was collected.

Eligibility criteria required radiologically confirmed degenerative L/S spinal stenosis, age between 18 and 80 years, failure of conservative therapy for at least six months, and the absence of contraindications to surgical or internal medical treatment. Additional inclusion criteria included the absence of hormonal or gastrointestinal disorders, and, in the case of female participants, not being pregnant or breastfeeding. The use of anticoagulants was restricted unless a medically approved discontinuation was possible. Subjects were excluded if they had a history of spinal surgery in the L/S region, lacked imaging-confirmed degeneration, had a successful outcome with conservative treatment, or exhibited any of the aforementioned contraindications. Intake of vitamin or mineral supplements classified as pharmaceutical supplements within six months prior to the study was also an exclusion factor.

### 2.2. Pain Assessment

Pain levels were evaluated using the Visual Analog Scale (VAS), a 10-point metric where 0 denotes no pain and 10 represents the most severe pain imaginable. No patients reported a pain below level 4. The distribution was as follows: 19 patients rated their pain as level 4, 22 at level 5, 23 at level 6, 9 at level 7, 8 reported levels 8–9, and 7 patients reported maximum pain at level 10.

### 2.3. Surgical Procedure

All patients underwent extended fenestration and foraminotomy under general anesthesia. A midline incision was made over the affected spinal level, and the paraspinal musculature was bluntly dissected to expose the posterior spinal elements. The hypertrophic ligamentum flavum was excised using Kerrison rongeurs. Decompression of the dural sac and exiting nerve roots was achieved via foraminotomy. Saline irrigation was used to clean the surgical field, and standard wound closure techniques were applied. Microsurgical techniques were used throughout the procedure. Patients without complications were generally discharged on postoperative day three and returned for outpatient follow-up approximately four weeks later.

### 2.4. Control Group

The control cohort included 85 individuals (39 females [46%] and 46 males [54%]), with a mean age of 49.17 ± 2.65 years. Ligamentum flavum tissue samples were collected postmortem during organ procurement procedures or forensic autopsies. Smoking status, alcohol use, and diabetic history were recorded when available, but detailed behavioral data were not included. Absence of degenerative changes was confirmed histologically via hematoxylin and eosin (H&E) staining. Two board-certified neurosurgeons independently verified each sample’s eligibility.

Control inclusion criteria included an age range of 18−80 years, no history of cancer or spinal trauma, no imaging evidence of spinal degeneration, and no recent intake of medication-classified dietary supplements. Subjects were also screened for the absence of endocrine or gastrointestinal pathologies. Exclusion criteria were aligned with those applied to the patient group, including pregnancy, lactation, supplement use, and systemic disease.

### 2.5. Sample Collection and Molecular Analysis

Ligamentum flavum samples were rinsed and preserved in sterile Eppendorf tubes containing RNAlater (Invitrogen, Carlsbad, CA, USA) to stabilize RNA. Samples were stored at −80 °C prior to molecular testing. Analyses included mRNA quantification, Western blotting, and ELISA. To prevent contamination, tissue collection was strictly limited to the ligamentum flavum, avoiding adjacent structures like the annulus fibrosus. Sample selection and anatomical precision were confirmed intraoperatively by two neurosurgeons (Dawid Sobański and Rafał Staszkiewicz).

### 2.6. Ribonucleic Acid (RNA) Isolation and Quality Control

Total RNA was extracted using the TRIzol reagent (Invitrogen, Carlsbad, CA, USA). Samples were homogenized using a T18 Digital Ultra-Turrax homogenizer (IKA Polska Sp. z o.o., Warsaw, Poland). Homogenates were mixed with TRIzol and chloroform (POL-AURA, Dywity, Poland), followed by centrifugation to isolate the aqueous RNA phase. RNA was then precipitated using isopropanol, washed in 70% ethanol, and treated with DNase I to remove genomic DNA. Final purification was performed with the RNeasy Mini Kit (Qiagen, Valencia, CA, USA). The purified RNA was air-dried and stored at −80 °C until analysis.

### 2.7. NRTN mRNA Analysis by Real-Time Polymerase Chain Reaction Technique Preceded by Reverse Transcription (RTqPCR)

Following RNA quality and quantity verification, RT-qPCR was conducted using specific primers (Genomed, Warsaw, Poland; Table 1). Glyceraldehyde-3-phosphate dehydrogenase (GAPDH) served as the endogenous control. Each biological sample underwent three technical replicates in a 50 µL reaction mixture. Amplification specificity was confirmed by determining the melting temperature (Tm) for each product. Gene expression changes were analyzed using the 2^−∆∆Ct^ method.

### 2.8. NTRT Protein Concentration Analysis via Enzyme-Linked Immunosorbent Assay (ELISA)

NRTN protein concentrations in ligamentum flavum samples were quantified using a commercial enzyme-linked immunosorbent assay (ELISA) kit (Human NRTN ELISA Kit, Abbexa, Cambridge, UK; catalog no. abx152513) following the manufacturer’s instructions. GAPDH protein expression (Santa Cruz Biotechnology, Dallas, TX, USA) served as an internal reference to normalize the results. The detailed ELISA procedures adhered to established protocols as outlined in our earlier work [18,19].

### 2.9. Western Blot

Ligamentum flavum tissues were harvested from both patient and control cohorts. Following thorough rinsing with phosphate-buffered saline (PBS), each tissue sample was placed into sterile microtubes and incubated in 0.5 mL of RIPA buffer (Sigma Aldrich, St. Louis, MO, USA) enriched with a protease and phosphatase inhibitor cocktail. Samples were mechanically homogenized using a T18 Digital Ultra-Turrax homogenizer (IKA, Warsaw, Poland) until fully lysed. After homogenization, samples were incubated on ice with gentle agitation for 60 min, followed by centrifugation. The resulting supernatants were collected and stored at −80 °C until further use.

Prior to electrophoresis, protein concentrations were determined using a bicinchoninic acid (BCA) assay (Thermo Fisher Scientific, Waltham, MA, USA) based on a standard curve prepared with bovine serum albumin (BSA) at six concentration points (0–2000 µg/mL; Sigma Aldrich). Protein extracts ranging from 20 to 100 µg were used for downstream analysis.

Western blotting was performed using 20 µg of total protein per sample, resolved on SDS-PAGE gels (POL-AURA, Dywity, Poland), and transferred to PVDF membranes with 0.45 µm pores (Thermo Fisher). Neurturin was detected using a polyclonal anti-NRTN antibody (bs-0073R, STI, Poznań, Poland; 1:300 dilution; expected molecular weight: 17 kDa). Beta-actin (ACTB; 1:500 dilution; Santa Cruz Biotech; MW: 37 kDa) was used as the loading control. A horseradish peroxidase-conjugated goat anti-rabbit IgG secondary antibody (Bio-Rad, Milan, Italy; 1:3000 dilution; catalog no. 1706515) facilitated chemiluminescent detection. Protein band intensity was quantified using Kodak MI 4.5SE software (Kodak, Rochester, NY, USA). Negative controls consisted of samples processed without the primary antibody, while HeLa cell lysates served as positive controls.

### 2.10. Immunohistochemical (IHC) Detection of NRTN

Paraffin-embedded ligamentum flavum tissues were sectioned at a thickness of 8 µm using a microtome (Leica Microsystems, Wetzlar, Germany). Standard immunohistochemical (IHC) procedures were performed, including deparaffinization, rehydration, antigen retrieval, and antibody incubations, based on manufacturer guidelines (Vector DAB Substrate Kit, Vector Laboratories, Newark, CA, USA; and IHC-Paraffin Protocol, Abcam, Cambridge, UK). Sections were incubated with the anti-NRTN polyclonal antibody (bs-0073R, STI, Poznań, Poland).

Visualization of immunoreactivity was performed using a Nikon Coolpix fluorescent microscope (Nikon Instruments Inc.; Melville, NY, USA). Quantitative image analysis was conducted using ImageJ software (version: 1.x) and the IHC-Profiler plugin. Fifteen representative images were collected from three stained slides per patient at 200× magnification. The percentage of DAB-positive areas was calculated relative to background staining, and optical density values were used to compare NRTN expression across samples.

### 2.11. Statistical Analysis

Statistical evaluations were conducted using dedicated analysis software. The normality of data distributions was verified using the Shapiro–Wilk test, while homogeneity of variance was assessed with Levene’s test. Continuous variables were reported as means with corresponding standard deviations (SD). Differences in mRNA and protein expression between patient and control groups were assessed using Student’s *t*-test. For comparisons across multiple subgroups (e.g., BMI categories and pain severity levels), a one-way ANOVA followed by Scheffé’s post hoc test was applied.

Linear regression analyses were used to examine potential predictors of NRTN expression, including age, BMI, smoking status, alcohol consumption, and diabetes. Correlations between NRTN levels and pain intensity (measured by VAS scores) were evaluated using the Pearson correlation coefficient (r). Multiple regression analyses were subsequently conducted to identify independent predictors of NRTN expression. Variables that did not reach significance in univariate models were excluded from the multivariate analysis. Model performance was assessed using adjusted R^2^ values. A *p*-value < 0.05 was considered statistically significant.

Subgroup analyses explored differences in NRTN expression by sex, BMI classification, smoking habits, alcohol intake, and diabetic status. Where appropriate, a two-way ANOVA was used to detect interactions between categorical variables.

## 3. Results

### 3.1. Expression Profile of NRTN at the mRNA Level 

*NRTN* mRNA expression was significantly upregulated—by 32.67 ± 2.98—in ligamentum flavum samples affected by degeneration relative to the control group.

### 3.2. Expression Profile of NRTN at the Protein Level in Test and Control Samples

#### 3.2.1. Concentration of NRTN Obtained via ELISA Assay and Western Blot Analysis

**Figure 1:**
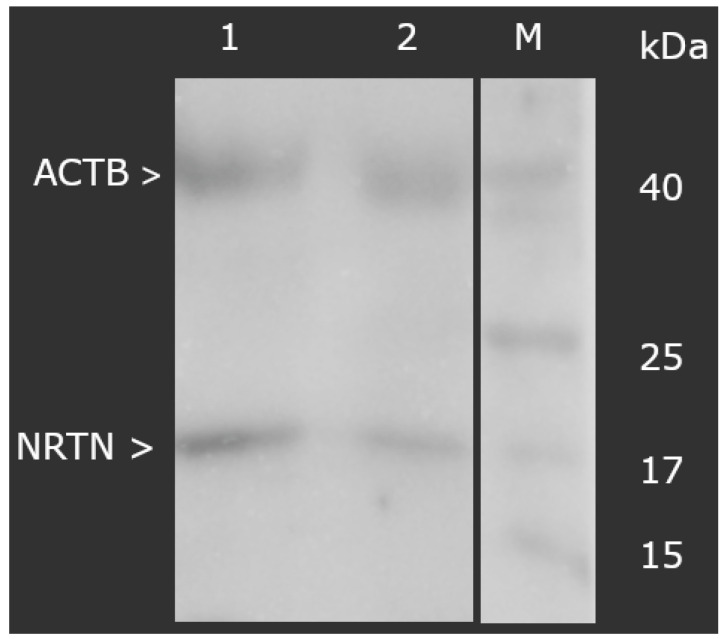
Normalized expression of NRTN in ligamentum flavum normalized against ACTB expression (Western blot). NRTN, neurturin; ACTB, beta-actin; M, molecular weight marker; 1, test group; 2, control group.

At the protein level, a markedly higher concentration of NRTN was also observed in the degenerated samples. ELISA testing revealed values of 12.34 ± 1.09 ng/mL in the study group versus 1.09 ± 0.16 ng/mL in controls (*p* < 0.05). Similarly, the Western blot analysis demonstrated that the normalized optical density of NRTN (molecular weight: 17 kDa), relative to ACTB, was significantly increased in the degenerated group (3.56 ± 0.19) compared to controls (0.76 ± 0.19), as shown in Figure 1 (*p* < 0.05).

#### 3.2.2. IHC Analysis

Furthermore, the IHC analysis confirmed the presence of NRTN in both the control and degenerated ligamentum flavum tissues, with visibly stronger staining in the pathological sections. For quantification purposes, the baseline optical density of NRTN in the control group was normalized to 100%. The degenerated samples exhibited a relative optical density of 191.76 ± 9.13%, indicating a 91.76 ± 9.13% increase in NRTN expression. In addition to optical density analysis, the percentage of the DAB-positive staining area was significantly greater in degenerated ligamentum flavum samples compared to controls (23.78 ± 4.5% vs. 12.4 ± 3.1%, *p* < 0.001, Student’s *t*-test).

**Figure 2:**
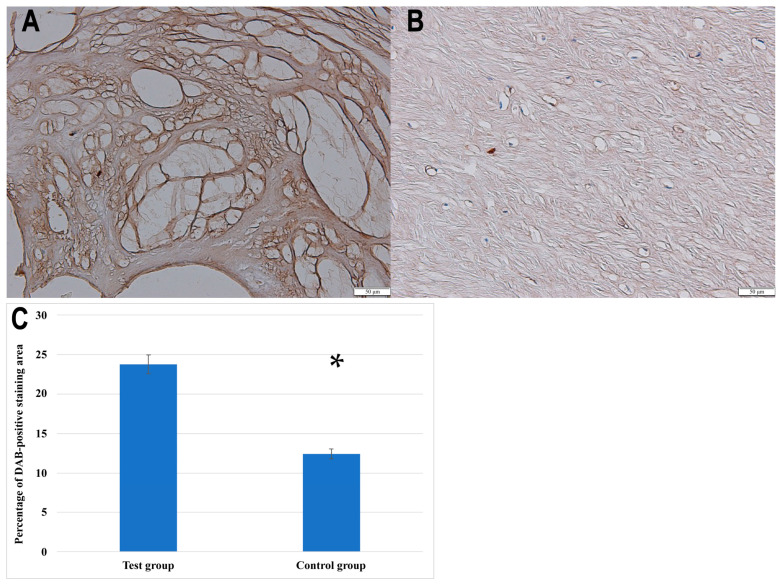
Immunochemical expression of NRTN in the test (**A**) and control (**B**) groups; (**C**) quantitative comparison of the percentage of DAB-positive staining area between the test group and the control group. Data are presented as mean ± SEM; *, statistically significance differences (*p* < 0.001).

Figure 2 presents representative immunohistochemical staining images of NRTN expression in ligamentum flavum tissues, along with a quantitative analysis of the DAB-positive staining area in the control and degenerated samples.

### 3.3. The Concentrations of mRNA and Protein of NRTN in the Tested Samples Depending on the Pain Degree Measured with the VAS

The analysis of NRTN levels in ligamentum flavum tissue demonstrated a progressive increase in both mRNA expression and protein concentration in relation to the severity of pain, as measured by the VAS scale (Table 2). As VAS scores increased from 2 to 10, mRNA expression rose from 27.84- to 36.94-fold, while protein levels increased from 9.91 to 13.56 ng/mL. The observed trend was statistically significant for both molecular parameters as confirmed by the one-way ANOVA (*p* = 0.032 for mRNA, *p* = 0.041 for protein).

### 3.4. Variances in the Expression Profiles of NRTN at the mRNA and Protein Levels in Ligamentum Flavum Samples Obtained from the Study and Control Groups

In the study group, *NRTN* mRNA and protein levels were moderately elevated in males compared to females, but the differences were not statistically significant (Table 3). Notably, both obesity and diabetes were associated with markedly increased levels of NRTN at both the transcript and protein levels. Protein levels ranged from 8.31 ng/mL in individuals with a normal BMI to 16.73 ng/mL in those with obesity (*p* < 0.0001). Similarly, diabetic patients exhibited higher NRTN protein levels (18.15 ng/mL) compared to non-diabetics (6.52 ng/mL).

Alcohol consumption was also associated with higher protein expression (13.59 vs. 11.08 ng/mL, *p* = 0.046), whereas smoking and sex had minimal impact on NRTN levels, with non-significant differences across groups (Table 3).

### 3.5. Regression Analysis of Variables Potentially Associated with NRTN Levels in Ligamentum Flavum Samples from the Study Groups

#### 3.5.1. Univariate Regression Analyses

The univariate linear regression revealed that several variables were positively associated with NRTN expression at both the mRNA and protein levels. Among them, BMI, diabetes, smoking, and alcohol consumption demonstrated moderate-to-strong associations (β ranging from 0.4 to 0.8), although not all associations reached statistical significance.

Interestingly, BMI and smoking showed the highest univariate β values (up to 0.8), suggesting a potential role in modulating NRTN levels in degenerative tissue. However, the lack of significance in some cases (e.g., BMI: *p* = 0.86 for mRNA) indicates variability or confounding by other variables. These univariate results served as the basis for variable inclusion in the multivariate model (Table 4).

#### 3.5.2. Multivariate Regression Analyses

Multivariate linear regression was performed to identify independent predictors of NRTN expression while controlling for covariates. Notably, BMI and diabetes remained statistically significant predictors of both mRNA and protein levels, with β values up to 0.50 (*p* = 0.015) for protein. This underscores their independent and robust association with NRTN expression, likely reflecting systemic metabolic influences on ligamentum flavum remodeling.

In contrast, while gender showed nominal associations in the univariate model, it did not remain significant after adjustment for other factors, suggesting a weaker or indirect effect.

The persistence of significance for smoking and alcohol consumption in the multivariate model supports the hypothesis that lifestyle-related oxidative or inflammatory stressors may upregulate neurotrophic signaling pathways in the ligament tissue. Table 5 presents the results from the multivariate analysis.

#### 3.5.3. Multivariate Regression Analyses of Variables Associated with NRTN Levels in Control and Study Group Ligamentum Flavum

To further delineate the impact of clinical and lifestyle factors on NRTN expression, we performed multivariate regression analyses stratified by group (control vs. study). This approach revealed that the strength of associations was consistently more pronounced in the degenerative group, suggesting a potential interaction between disease state and the examined variables. BMI emerged as the strongest independent predictor of NRTN protein levels in the study group (β = 0.50, *p* = 0.015) compared to a much weaker association in the control group (β = 0.13, *p* = 0.049). A similar pattern was observed for diabetes (study: β = 0.39, *p* = 0.017 vs. control: β = 0.11, *p* = 0.056) and smoking (study: β = 0.38, *p* = 0.020 vs. control: β = 0.15, *p* = 0.051). The results of the multivariate regression analyses of variables associated with NRTN levels in control and study group ligamentum flavum are presented in Table 6.

#### 3.5.4. Multivariate NRTNT Protein Levels: Impact of Pain Severity and Lifestyle Factors

In the final multivariate model summarizing pain and lifestyle factors, VAS pain score, BMI, diabetes, smoking, and alcohol use all showed positive and statistically significant associations with NRTN protein levels. Among these, BMI (β = 0.50, *p* = 0.015) and diabetes (β = 0.39, *p* = 0.017) remained the strongest independent predictors, reinforcing their relevance to the neurotrophic profile of ligamentum flavum in degenerative spinal disease (Table 7). These results suggest a synergistic effect of pain intensity and metabolic/inflammatory status on NRTN expression. The inclusion of VAS as a significant predictor underscores the potential of NRTN as a molecular correlate of pain severity in degenerative spinal conditions.

Together, these findings provide compelling evidence that NRTN levels in ligamentum flavum are modulated by both systemic (e.g., diabetes and obesity) and behavioral (e.g., smoking and alcohol) factors, with a potential link to clinical pain outcomes.

## 4. Discussion

Despite growing interest in the molecular aspects of spinal degenerative pathologies, the role of NRTN in ligamentum flavum remodeling and pain modulation in lumbar spinal stenosis remains poorly understood. Previous studies have predominantly focused on classical neurotrophins such as nerve growth factor (NGF) and brain-derived neurotrophic factor (BDNF), often overlooking glial cell line-derived neurotrophic factor (GFL) family members—NRTN included—in translational research [18,19,20], while members of the GDNF family, including NRTN, have received comparatively little attention [21,22]. This study addresses that gap by evaluating NRTN expression in ligamentum flavum tissue and its relationship with pain severity, metabolic risk factors, and lifestyle behaviors.

Our findings demonstrate a significant upregulation of NRTN at both the mRNA and protein levels in degenerative ligamentum flavum tissue compared to non-pathological controls, supported by results from RT-qPCR, ELISA, Western blotting, and IHC. Importantly, NRTN levels increased in parallel with VAS pain scores, implicating this neurotrophic factor in nociceptive processes within the stenotic spine. Multivariate regression analyses revealed that among several clinical variables, BMI and diabetes were the strongest independent predictors of increased NRTN expression. These associations suggest that chronic systemic conditions may influence the local microenvironment of the ligamentum flavum, possibly through metabolic and inflammatory pathways.

NRTN, a member of the glial cell line-derived neurotrophic factor family, is known for supporting neuronal survival and repair. Under normal physiological conditions, its expression is limited; however, it can be upregulated in response to tissue injury, hypoxia, or inflammation [12,13]. In the context of LSS, where ligamentum flavum thickening leads to compression and microinjuries of neural structures, elevated NRTN may reflect an adaptive response to ongoing mechanical stress and nerve root irritation. Analogous to proteins like GAP-43, which are implicated in neuroplasticity and axonal regeneration, NRTN may contribute to restoring homeostasis by promoting peripheral nerve repair [12,21,23,24,25,26]. The observed associations between NRTN expression and metabolic factors such as obesity and diabetes further support this hypothesis. Both conditions are known to promote systemic inflammation, oxidative stress, and neurovascular dysfunction [27,28]. These mechanisms may be protective initially, but prolonged upregulation of neurotrophic signaling could contribute to maladaptive plasticity and persistent pain. The ligamentum flavum, as a vascularized and innervated structure, may respond to these systemic influences by activating local neuroprotective signaling pathways.

Although we also observed higher NRTN expression in patients reporting alcohol consumption and smoking, these variables did not remain independent predictors in multivariate analysis. This suggests that their effects on NRTN may be indirect—possibly mediated by systemic inflammation or overlapping metabolic disturbances. Similar patterns have been reported for other neuroplasticity-associated proteins, including growth associated protein (GAP-43) [29,30]. Notably, the potential role of NRTN in neuroplasticity and nociception remains speculative. While elevated levels may support neural regeneration, chronic overexpression could, paradoxically, promote hyperalgesia or persistent pain. This dual role has been proposed for other neurotrophins in chronic pain syndromes and warrants further investigation [31,32,33]. A recognized limitation of this study is the use of postmortem tissue as a control source, which may raise concerns about molecular integrity. While this approach is common in neurodegenerative and spinal pathology research, potential confounding effects include RNA and protein degradation, as well as premortem systemic conditions that could affect gene expression profiles.

To mitigate these risks, all postmortem samples were obtained within a controlled time window after death and immediately preserved in RNA stabilization reagents (e.g., RNAlater), which are well-documented to maintain RNA and protein integrity in unfrozen tissue [15,34,35,36,37,38,39,40,41,42]. Additionally, tissues were handled under standardized, preanalytical protocols to minimize degradation artifacts.

Importantly, no significant differences in reference genes such as GAPDH and beta-actin (ACTB) were observed between control and degenerative groups, supporting the overall molecular comparability of the samples.

Nevertheless, it must be acknowledged that premortem systemic conditions (e.g., undiagnosed diabetes, inflammation, or subclinical infections) may have affected the baseline expression levels in postmortem controls. These unknowns underscore the need for cautious interpretation and further validation of findings in prospectively collected surgical samples, where ethically feasible.

Future research should focus on several key areas. First, longitudinal studies are warranted to monitor changes in NRTN expression over time and their association with ligamentum flavum hypertrophy and clinical symptom progression. Second, the role of lifestyle factors and comorbidities in modulating NRTN expression should be further elucidated to identify potential therapeutic intervention targets. Finally, functional studies aimed at understanding how NRTN influences neuroplasticity and pain modulation could pave the way for novel therapeutic strategies in the management of spinal canal stenosis.

## 5. Conclusions

This study identifies NRTN as a potentially important mediator in the pathophysiology of lumbar spinal stenosis. Its elevated expression in hypertrophied ligamentum flavum correlates with pain intensity and metabolic disturbances, particularly obesity and diabetes. These findings suggest that NRTN may reflect a compensatory response to chronic mechanical and metabolic stress. Further investigation is needed to clarify whether NRTN plays a protective, maladaptive, or dual role in spinal degeneration and whether it can be harnessed for therapeutic purposes.

## Figures and Tables

**Table 1:** Primer sequences for RT-qPCR.

mRNA	Oligonucleotide Sequence
*NRTN*	Forward: 5′-CTGCCTGTGATGCCATTCTC-3′ Reverse 5′-GCCTTTGACTTTGAACGCCT-3′
*GAPDH*	Forward: 5′-GGTGAAGGTCGGAGTCAACGGA-3′ Reverse 5′-GAGGGATCTCGCTCCTGGAAGA-3′

Forward, sensible starter; reverse, antisense primer; *GAPDH*, dehydrogenase 3-phosphoglyceraldehyde; *NRTN*, neurturin.

**Table 2:** Expression levels of NRTN mRNA and protein concentration in ligamentum flavum tissue from patients with lumbar spinal degeneration, stratified by pain severity (VAS scale).

VAS Pain Intensity	*NRTN* mRNA Expression (Fold Change ± SD)	NRTN Protein Concentration (ng/mL ± SD)	ANOVA (*p*)
2	27.84 ± 0.48	9.91 ± 0.34	0.032 ^a^ 0.041 ^b^
3	29.93 ± 0.39	10.57 ± 0.31	
4	31.18 ± 0.52	11.23 ± 0.36	
5	32.42 ± 0.47	11.77 ± 0.44	
6	32.76 ± 0.40	12.19 ± 0.42	
7	33.64 ± 0.38	12.49 ± 0.39	
8	34.47 ± 0.33	12.86 ± 0.49	
9	35.73 ± 0.42	13.17 ± 0.51	
10	36.94 ± 0.58	13.56 ± 0.60	

Data are presented as mean ± SD. A one-way ANOVA was used to assess the significance of the trend across VAS groups; ^a^, *p*-value for ANOVA (mRNA level); ^b^, *p*-value for ANOVA (protein level).

**Table 3:** The expressions of NRTN at the mRNA and protein levels in ligamentum flavum samples obtained from the study group.

Comparison		mRNA	Student’s *t*-Test ^1^ or ANOVA ^2^ (Study Group)	Protein	Student’s *t*-Test ^1^ or ANOVA ^2^ (Control Group)
Gender	Female (n = 43)	31.87 ± 1.67	0.761 ^1^	11.97 ± 1.03	0.651 ^1^
	Male (n = 38)	33.47 ± 1.84		12.71 ± 0.77	
BMI (kg/m^2^)	Normal (n = 54)	1.00	0.013 ^2^	8.31 ± 0.76	< 0.0001 ^2^
	Overweight (n = 42)	26.12 ± 2.34		11.97 ± 0.66	
	Obesity (n = 17)	39.19 ± 3.18		16.73 ± 0.57	
Diabetes	No (n = 91)	27.54 ± 2.52	0.024 ^1^	6.52 ± 0.87	< 0.0001 ^1^
	Yes (n = 22)	37.39 ± 1.87		18.15 ± 1.23	
Smoking	No (n = 77)	32.37 ± 1.34	0.819 ^1^	12.29 ± 0.54	0.742 ^1^
	Yes (n =36)	32.29 ± 2.19		12.39 ± 0.81	
Drinking alcohol	No (n = 8)	31.17 ± 2.11	0.438 ^1^	11.08 ± 0.44	0.046 ^1^
	Yes (n = 105)	34.16 ± 1.48		13.59 ± 1.13	

Data are presented as means and standard deviations. BMI, body mass index.

**Table 4:** Univariate regression analyses of the variables that may be associated with NRTN levels determined in the degenerative ligamentum flavum.

Variable	Expression Type	Univariate β	Univariate *p*-Value
Gender	mRNA	0.200	0.010
	Protein	0.170	0.010
BMI	mRNA	0.800	0.860
	Protein	0.770	0.800
Diabetes	mRNA	0.550	0.380
	Protein	0.550	0.370
Drinking alcohol	mRNA	0.520	0.200
	Protein	0.400	0.220
Smoking	mRNA	0.790	0.440
	Protein	0.800	0.530

BMI, body mass index. Variables found to be insignificant using linear regression were not included in the multiple regression model.

**Table 5:** Multivariate regression analyses of the variables that may be associated with NRTN levels determined in the degenerative ligamentum flavum.

Variable	Expression Type	Multivariate β	Multivariate *p*-Value
Gender	mRNA	0.430	0.410
	Protein	0.480	0.410
BMI	mRNA	0.000	0.019
	Protein	0.000	0.022
Diabetes	mRNA	0.000	0.018
	Protein	0.000	0.022
Drinking alcohol	mRNA	0.032	0.018
	Protein	0.030	0.021
Smoking	mRNA	0.010	0.023
	Protein	0.004	0.030

BMI, body mass index. Variables found to be insignificant using linear regression were not included in the multiple regression model.

**Table 6:** Multivariate regression analyses of variables associated with NRTN levels in control and study group ligamentum flavum.

Characteristic	Expression Level	Control Group		Study Group	
		Coefficient	*p*-Value	Coefficient	*p*-Value
Gender	mRNA	-	-	-	-
	Protein	-	-	-	-
BMI (kg/m^2^)	mRNA	0.11	0.062	0.47	0.013
	Protein	0.13	0.049	0.50	0.015
Diabetes	mRNA	0.09	0.071	0.36	0.014
	Protein	0.11	0.056	0.39	0.017
Smoking	mRNA	0.13	0.065	0.34	0.019
	Protein	0.15	0.051	0.38	0.020
Drinking Alcohol	mRNA	0.07	0.081	0.18	0.017
	Protein	0.09	0.074	0.21	0.018

BMI, body mass index.

**Table 7:** Summary of multivariate regression analysis on NRTN protein levels in relation to pain and lifestyle factors.

Factor	Association with NRTNT (Univariate)	*p*-Value (Univariate)	Coefficient in Multivariate Model	*p*-Value (Multivariate)
VAS Pain Score	Positive	0.032	0.31	0.017
BMI	Positive	<0.0001	0.50	0.015
Smoking	Positive	0.004	0.38	0.020
Alcohol Consumption	Positive	0.03	0.21	0.018
Diabetes	Positive	<0.0001	0.39	0.017

## Data Availability

The data used to support the findings of this study are included in the article. The data cannot be shared due to third-party rights and commercial confidentiality.
